# Design of Docking Interfaces for On-Orbit Assembly of Large Structures in Space

**DOI:** 10.3390/s24206534

**Published:** 2024-10-10

**Authors:** Shuai Liu, Enyang Zhang, Zhenbang Xu, Jingxu Zhang

**Affiliations:** 1 Changchun Institute of Optics, Fine Mechanics and Physics, Chinese Academy of Sciences, Changchun 130033, China; ls897714447@163.com (S.L.); xuzhenbang@ciomp.ac.cn (Z.X.); zhangjingxu@ciomp.ac.cn (J.Z.); 2 University of Chinese Academy of Sciences, Beijing 100049, China; 3 Key Laboratory of On-Orbit Manufacturing and Integration for Space Optics System, Chinese Academy of Sciences, Changchun 130033, China

**Keywords:** docking interface, hermaphrodite, misalignment, on-orbit assembly, modularity

## Abstract

Considering the complexity of on-orbit assembly during space missions and the super-large size of space structures, this paper presents the design for a new type of docking interface with an androgynous body that exhibits a number of advantages, including high connection strength and a compact structure. The androgynous body has a conical guided symmetric design with a symmetry of 90°. The geometric design of the docking surface is described in detail in order to prove its advantages. Structural design was carried out using UG modeling as well as dynamic simulation using Recur Dyn to obtain the displacement coordinate curves of the docking port. The geometry of the docking port’s high docking misalignment tolerance was verified, and misalignment tolerance and lens splicing experiments were also performed. The docking port’s ability to be quickly connected or disconnected within a translation tolerance of 23.5 mm and a tilt tolerance of 24° was verified. This article provides a useful reference for space missions in terms of module docking and on-orbit assembly.

## 1. Introduction

With the development of aerospace, the demand for large space structures, such as space stations, space telescopes and large communication antennas, has gradually increased. Space missions in orbit are gradually becoming more and more complex, and existing space structures are built and maintained by robotic arms. For example, the tasks of space station construction, module segment docking, in-orbit maintenance and replacement, as well as the robotic arm’s self-reconfiguration [1], and the end-tools quick-exchange, have all generated higher docking interface technologies requirements.

The performance of docking interfaces plays a decisive role in space on-orbit missions as well as the self-reconfiguration of robotic arms [2]. The existing docking interfaces are unidirectional, distinguishing between active and passive interfaces [3], and have small docking misalignment tolerances, making it difficult to carry out the assembly of ultra-large structures in space as well as more complex on-orbit missions. For this reason, this paper proposes an androgynous mold docking interface, which adopts a compact, fully integrated androgynous and 90-degree symmetric design, and does not distinguish between active and passive interfaces in the docking process. In this design, a blocking ball is used for the interface’s locking functionality, which has the advantages of high connection strength, a compact structure and high misalignment tolerance [4].

The main design purpose for a self-reconfiguring robot is its reconfiguration connection mechanism, and the main basis for the overall self-reconfiguration motion of the system is a flexible and reliable docking mechanism [5]. The docking mechanism plays a vital role in allowing the modular robot to self-repair and self-reconfigure, which facilitates the operation of the docking mechanism with high efficiency, uniformity and high misalignment tolerance.

J Jiang Dongsheng [6] and others proposed a novel spatial self-reconfigurable modular robot, M-Lattice, which has a special docking mechanism based on pin-axis wedging and androgyny. The consistency of the structure of the mating surfaces ensures that the pins on both mating surfaces can be inserted into each other’s ring grooves at the same time. Precise wedging between the mating surfaces is achieved by the sliding of the pins and the annular grooves, which complete the modular connection. When disengagement is required, it is only necessary to reverse the docking motor. Typical representatives of this type of mechanical interface are, for example, the JL-2 robot of the Beijing University of Aeronautics and Astronautics [7], which adopts a mechanical gripper for inter-module connection, and the reconfigurable robot designed by Sohal et al. [8], which adopts a clamping interface. The reconfigurable crawler robot designed by Xudong Zhang [9] and Chenglin He [10] uses a gripper docking mechanism, whereby docking occurs through a jaws and rings design; the jaws open and close via an electromagnet, which in turn connects with the rings to allow for the docking of the robot. USC’s Superbot module uses a multi-slider structure to simulate a “hand grasp” for connection and separation [11]. The HitMSR designed by Jie Zhao et al. [12] uses an electromagnetic interface. The M-TRAN and M-TRAN II proposed by the AIST Institute [13,14] use electromagnetic interfaces for docking and separation between modular robots by means of magnets as well as shape memory alloys. Chuanwu Zhao [15] employs neodymium magnets for the docking of modular robots.

Jian Chen et al. [16] set out to propose a new genderless docking system with a structural design characterized by a passive locking and single-sided electromagnetic unlocking mechanism. The axial locking mechanism uses a rotating plate with semicircular hooks and grooves to connect two opposing interfaces. Frictional anti-torque between the housing and the circumferential locking element is also used to constrain the rotation of the rotating plate. SINGO utilizes four translating jaws to engage or disengage the four jaws of the antagonistic mechanism, which can provide a certain degree of misalignment tolerance, but the time of the action is limited by the jaws’ movement speed and distance. GENFA [17] uses a fixed disk and a rotating disk with four chamfered pins and chamfered holes to constrain the rotational and axial degrees of freedom, respectively. However, the angular and translational errors were limited by the radius of the chamfer pins and holes. RoGenSiD [18] used a rotating plate with four androgynous locking fingers to engage the curved profile of the fingers in another connector. To prevent accidental separation along the long axis, alignment stakes were added, but this resulted in interfaces that were not strictly genderless. HiGen [19] and the Omni-Pi-tent connection system [20] are similar in design to RoGenSiD. HiGen utilizes a shield that translates along the axis to screw the hooks into and out of the docking interface in approximately 0.2 s of the connecting or disconnecting motion. The Omni-Pi-tent docking system has guide pegs and guide holes around the center of rotation to improve error tolerance. GHEFT [21] utilizes an h-slot clamping profile to provide a high misalignment tolerance and to obtain high clamping force and torque. The docking mechanism, which is exposed outside the housing after docking, should be improved for safety reasons.

End-effector models include the Canadian SRMS end-effector, SSRMS end-effector (LEE) and its target adapter (PDGF), the Japanese JEMRMS end-effector, the European Space Agency (ESA) ERA end-effector [22,23,24], the Chinese Space Station Core Module Mechanical Arm (CSSCMM) end-effector [25] and the Chinese Space Station Experiment Module Mechanical Arm (CSSEMM) end-effector [26]. The above end-effectors basically adopts three schemes: the steel wire-cone rod capture scheme (SRMS, SSRMS, JEMRMS, etc.), the three-pronged table guiding surface-capture hook scheme (ERA) and the three-finger-three-flap capture scheme (CSSEMM). In response to the needs of small and medium-sized space robots, a miniaturized and lightweight end-effector was developed at home and abroad. NASA designed an end-effector for Robonaut2 in 2013, which can assist R2 to complete climbing by grasping the handrail [27]. Japan’s GITAI company designed an end-effector for the IN1 robot in 2022, which is equipped with an internal camera and light source and was capable of completing geometrical climbing, tool replacement and other tasks in a lunar environment simulation test [28]. The Shanghai Institute of Aerospace Systems Engineering designed a self-moving robotic arm end-effector that exhibits miniaturization, large tolerance and high stiffness features that met the needs of a robotic arm “transposition walking” task [29].

In addition, for modular structure in-orbit assembly, robotic operation and other needs, the domestic and foreign development of a variety of standardized interfaces can also realize the function of the end-effector. iSSI, an intelligent space system interface developed by iBOSS in 2017, has mechanical, electrical, data, and thermal coupling functions; it also has the smallest mass and volume among the many interfaces [30,31]. HOTDOCK is a standardized interface designed by Space Application Services of Belgium. This interface provides mechanical connection, data communication, power delivery and heat transfer. It utilizes a four-guide flap heterogeneous homogeneous docking surface with high tolerance and docking flexibility [32,33]. Spain’s SENER Aeroespacial et al. designed the standard interface SIROM for robotic operation in 2018, which has a fluid transfer interface reserved on the basis of the above four functions [34].

**Figure 1:**
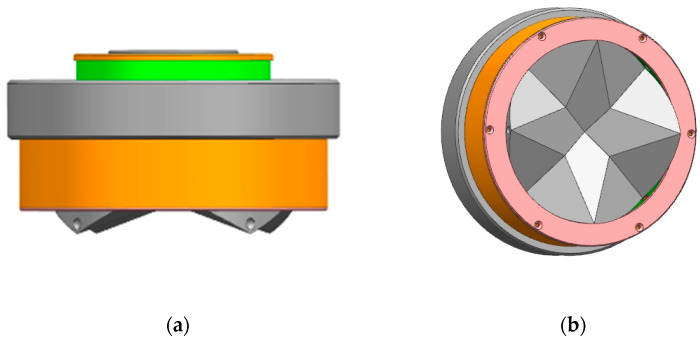
(**a**) Front view of interface (**b**) Interface Axonometric Drawing.

This paper develops an androgynous mating interface with a compact, fully integrated androgynous and 90-degree symmetric design, as shown in Figure 1. The design’s external geometry supports a high-tolerance tapered mating trajectory for the simultaneous coupling of three orthogonally mounted interfaces. The unique coupling mechanism along the circumference enables rigid mechanical structure coupling for high load transfer. The docking interface is one of the key components of reconfigurable robots and space-on-orbit assemblies, and is closely related to the design of the inter-module docking interface for the connection and controlled separation of robotic modules during reconfiguration, as well as for the robust connection of the space-on-orbit replacement unit modules. The docking interface thus plays a crucial role in realizing the space-on-orbit mission.

In this paper, we focus on designing a docking interface that has a high docking tolerance and is hermaphroditic without distinguishing between active and passive ends, which is highly adaptable and can be applied in multiple fields and contexts.

The rest of the paper is organized as follows: Section 1 describes the structure and working process of the butt joint interface. The misalignment tolerance of the docking interface is calculated in Section 2. The misalignment tolerance results from the simulation are shown in Section 3 and experimental verification is carried out in Section 4. This paper’s conclusion and proposed avenues for future work are discussed in Section 5.

## 2. The Structure and Working Process of the Butt Joint Interface

**Figure 2:**
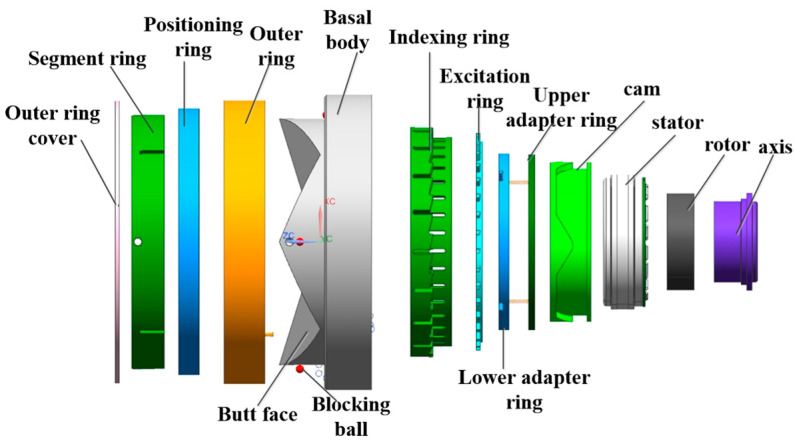
Exploded view of interface part structure.

**Figure 3:**
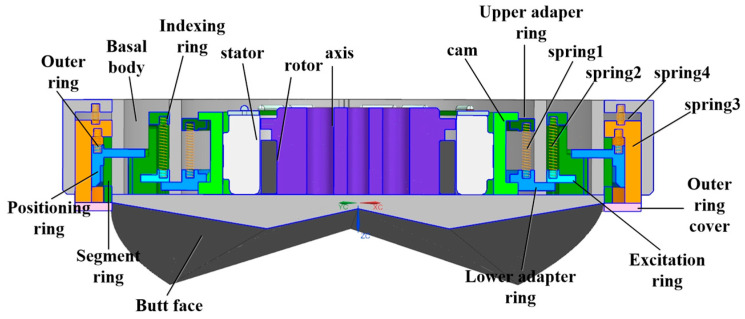
Structural section view of interface parts.

The three-dimensional structure of the docking interface is shown in Figure 2 and Figure 3; the main components are cam, rotor, stator, upper adapter ring, lower adapter ring, indexing ring, excitation ring, outer ring and positioning ring. The interface movement process mainly includes the docking and locking processes; the docking process is mainly guided by the interface docking surface for positioning, and the locking mechanism is locked by the blocking ball after the docking is completed.

### 2.1. Docking Process

The two interfaces are equipped with interface 1 and interface 2 by means of a robotic arm during the docking movement. The robotic arm is equipped with a six-dimensional force/torque sensor between the robotic arm and interface 1, which analyzes the suppleness of the docking process and the success of the docking by the change of force.

The blocking ball is in the gap between the separating ring and the positioning ring, and is free to move radially in the gap; during the docking of the two interfaces, the blocking ball of interface 1 will enter the corresponding groove of the docking surface of interface 2 under the action of the positioning ring.

### 2.2. Locking Process

**Figure 4:**
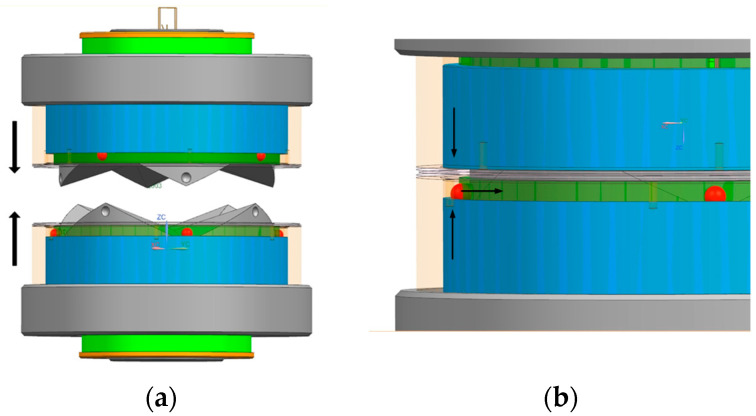
(**a**) Diagram of interface docking and locking process. (**b**) Partial enlargement of the interface docking and locking.

After the docking movement is completed, the locking mechanism of interface 1 drives the cam to carry out a clockwise rotary movement under the action of the motor, the upper adapter ring and the lower adapter ring make contact with the cam contour line under the force of spring 1, the lower adapter ring moves downward in the guiding groove of the interface substrate along the cam contour line and, in the process of moving upwards, the upper locating ring makes contact with the excitation ring through the guiding groove and pushes upward against the excitation ring. The excitation ring is preloaded with spring 2 between the excitation ring and the indexing ring, the excitation ring moves in the guiding groove of the indexing ring, and during the upward movement it will resist the positioning ring and push the positioning ring upward, the excitation ring and the positioning ring are contacted by the beveled surface, the indexing ring rotates counterclockwise by a certain angle under the action of spring 3 and spring 4 and the positioning ring then moves from the original short groove of the indexing ring to the long groove of the indexing ring. Under the action of spring 4, the outer ring moves to the locking position and, under the action of spring 3, the positioning ring moves to the end to squeeze the blocking ball. The radial partial force generated causes the blocking ball to move to the spherical groove in the docking surface of interface 2, and the positioning ring moves directly to the lowest end and, under the action of the spring, it is locked. This process is shown in Figure 4.

## 3. Structural Design and Tolerance Analysis of Docking Surfaces

### 3.1. Design of Buttressing Surface Parameters

**Figure 5:**
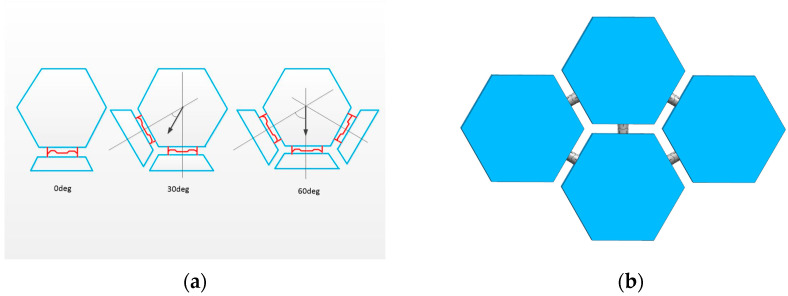
(**a**) Schematic diagram of hexagonal adaptation of interface butt face; (**b**) Schematic diagram of space-assembled lenses in orbit.

In order to make the interface more adaptable as well as efficient, the interface is designed with an androgynous structure that couples with the same interface. It also has a 90° mechanical symmetry, which allows for different orientations of the payload and simpler robotic maneuvers to prepare the coupling. The design of the interface’s docking surface geometry, for the space telescope lens on-orbit assembly mission, allows for the simultaneous matching of interfaces exposed in a hexagonal configuration, permitting the simultaneous coupling of three vertically mounted interfaces, as shown in Figure 5.

**Figure 6:**
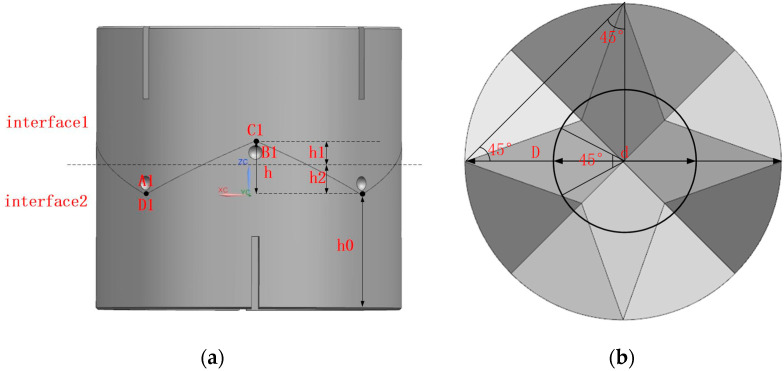
(**a**) Shape parameters of the left view of the interface butt face; (**b**) Shape parameters of interface butt face top view.

The design of the interface’s docking surface parameters is shown in Figure 6. In order to complete the task of assembling lens splicing in orbit, three hexagonal interfaces are docked at the same time. The formula for this is as follows:

h2 <= dsin30°/2cos30°(1)

D is the diameter of the interface, h2 is the fit height above the interface docking surface, h1 is the fit height below the docking surface and d is the diameter of the middle circle of the structural design. h = h1 + h2,h is the overall fit height of the conical block. A1 is the apex of the conical block of interface 1, C1 is the lowest apex of the concave surface of interface 1, B1 is the apex of the conical block of interface 2 and D1 is the lowest apex of the concave surface of interface 2. The lowest point of interface 1 and the highest point of interface 2 coincide with the highest point of interface 1 after the interface is docked. D1 is the lowest vertex of concave surface for interface 2.

**Figure 7:**
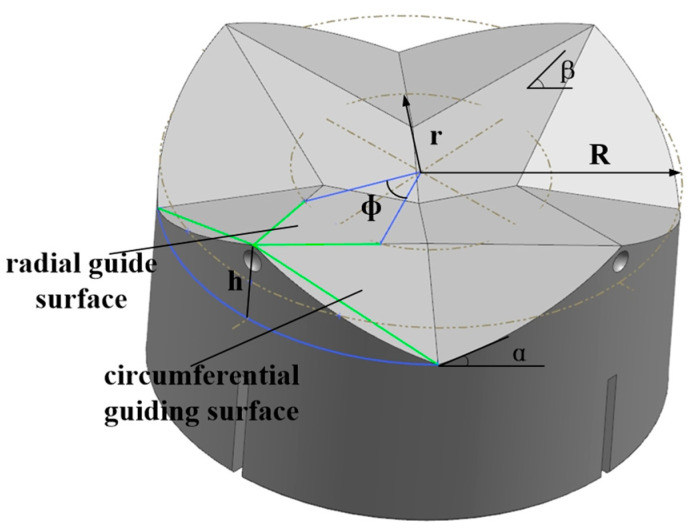
Docking Interface Geometry.

In the docking process, there will be a certain amount of positional error on both sides of the docking, due to factors such as robot positioning error. In order to ensure that the docking module has enough tolerance capacity to correct the positional error, its tolerance capacity was analyzed and its geometric parameters were optimized, as shown in Figure 7.

R is the radius of the outer ring of the guide ring, r is the radius of the inner ring of the guide ring, β is the radial guiding angle, α is the circumferential guiding angle and its expression is:

α = arctan(4h/πR)(2)

First of all, the successful docking of two docking modules needs to satisfy the axial projection of the tops of any two guiding flaps of docking interface 1, which is not simultaneously located in the radial guiding surface of the guiding flaps of the docking interface, according to which the radial tolerance difference region is calculated as the approximate internal tangent circle radius R_C_:

R_C_ = R cos(π/4) − r sin(Φ/2) [R − R sin(π/4)]/[R − r cos(Φ/2)] (3)

In this formula, Φ for the guide flap radial guide face should be a centered angle. The formula is:

Φ = π/4 − [h − 2tanβ(R − r) − h]/tan(αr)(4)

In addition, in order to ensure that the docking module can slide relative to each other under the action of axial force and avoid frictional self-locking, let the static friction factor of the contact surface be μ, then it must satisfy the following:

α,β > arctan μ(5)

Based on the above tolerance and mechanical conditions, an optimized design of geometric parameters of docking module was carried out. Take radial guiding angle β = 18°. According to the dimensions of the locking module and the electric connection module, reserve enough internal space, take the inner circle radius of guide ring r = 30 mm, under the premise of meeting the requirements of tolerance, so that the structure size is minimized, then take the outer circle radius of guide ring R = 40 mm, take the height difference h = 14 mm, and check the circumferential guiding angle α = 24.5, and finally check the radial tolerance radius R_C_ = 25 mm, which meets the requirements of the design indexes.

### 3.2. Analysis of Butt Joint Misalignment Tolerance

**Figure 8:**
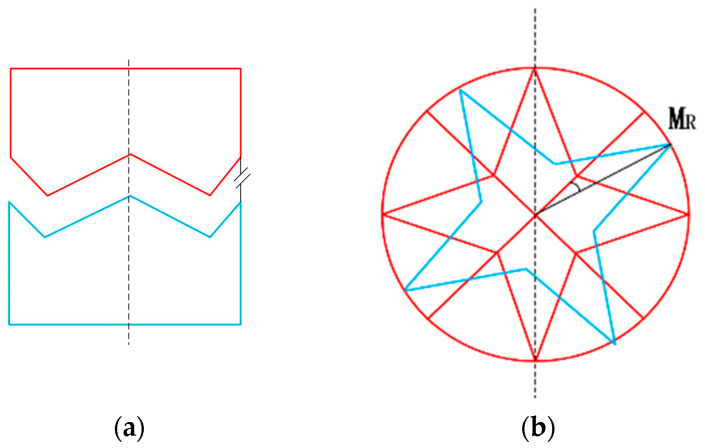

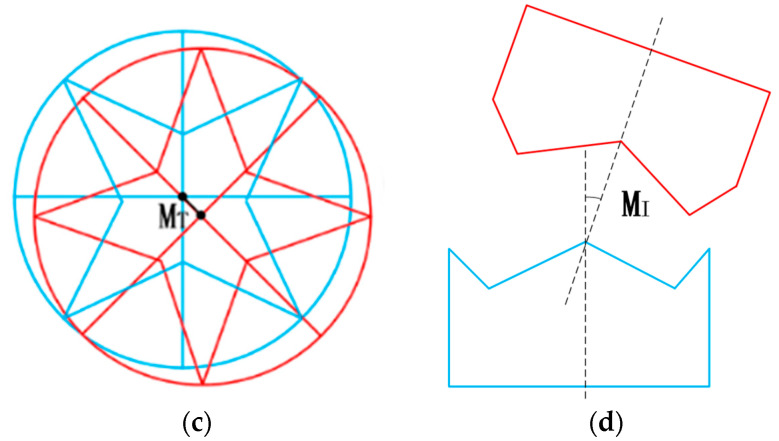
Interface misalignment tolerance analysis; (**a**) Parallel docking of the two interfaces; (**b**) Rotational misalignment tolerance; (**c**) Translational misalignment tolerance; (**d**) Inclination misalignment tolerance.

Compliance of the docking mechanism is necessary to ensure the success rate of docking. The alignment of the guided structure is similar to the pinned hole task [35]. Interface misalignment tolerances are categorized into three main types: rotational misalignment tolerance, translational misalignment tolerance, and inclination misalignment tolerance. These are illustrated in Figure 8.

#### 3.2.1. Rotational and Translational Misalignment Tolerances

**Figure 9:**
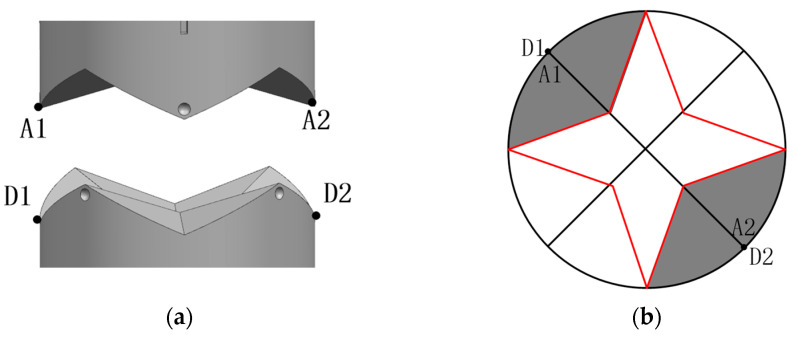
(**a**) 3D docking model; (**b**) 3D model projection.

The rotational and translational misalignment tolerances were analyzed in the case where the two interfaces were parallel (see Figure 8a). As shown in Figure 8b, when the axes of the two docking mechanisms coincide, the conical tops of the two docking interfaces happen to collide and fail to complete the docking when the misalignment tolerances of the two interfaces reach 90 degrees during docking. Such a situation can be neglected in practice. Therefore, the conical top of the docking surface ensures a successful connection with any rotational misalignment tolerance, MR [16]. The translational misalignment tolerance, MT, exists between the two axes of the mating docking mechanism, as shown in Figure 8c. Due to the symmetry of the structure, the four conical blocks are divided into two pairs parallel to each other. Taking one of the pairs of conical blocks as an example for analysis, the adaptive translational motion is guided by the top bevel of the conical blocks including the conical edges (see Figure 9). The projection of Figure 9a on the interface is shown in Figure 9b. In order to ensure that the tapered block can be inserted into the corresponding tapered concave surface of the interface and the projection of the A1–A2 vertices SP (vertices) on the interface needs to lie within the projection of the tapered concave surface SP (concave surface) defined by the relative tapered vertices, D1–D2. The above relationship is described as follows:

Sp(A) ∈ Sp(D)(6)

#### 3.2.2. Translational Misalignment Tolerance Analysis

**Figure 10:**
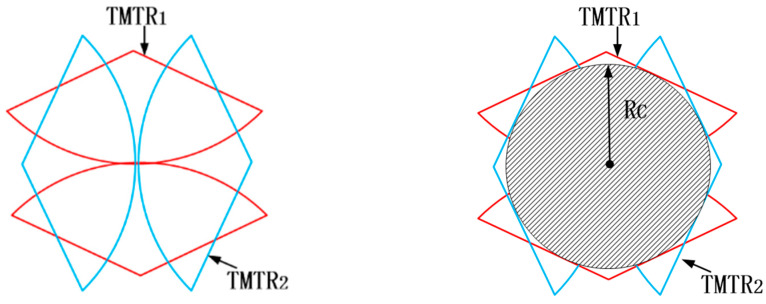
Theoretical diagram of translational misalignment tolerance for butt joint of two interface.

The translational misalignment tolerance range (TMTR) of a single pair of tapered blocks is shown as the center contour line in Figure 9b. The edges of the TMTR surround the common TMTR of the two vertices A1 and A2. The constraints between the two pairs of tapered blocks compensate each other during the alignment process. As long as one pair of trapezoidal blocks is located on the corresponding TMTR, the other two pairs of tapered blocks can be guided to their TMTRs, even though they are initially located outside their TMTRs. This means that only one pair of tapered blocks needs to be placed in the corresponding TMTR before docking. As shown in Figure 10, the two pairs of tapered blocks, TMTR_1_~TMTR_2_, are symmetrically distributed at 180° intervals along the rotation axis. The total translational misalignment tolerance range, TMTR_t_, for TMTR_1_~TMTR_2_ is defined as:

TMRTt = TMRT1∪TMRT2(7)

As is shown in Equation (3), TMTRt is the merger of TMTR1~TMTR2. The maximum internal tangent circle of the contour line is taken as the theoretical TMTRt of the two mating mechanisms. The radius of the maximum internal tangent circle is RC, as shown in Figure 10.

#### 3.2.3. Camber Misalignment Tolerance Analysis

**Figure 11:**
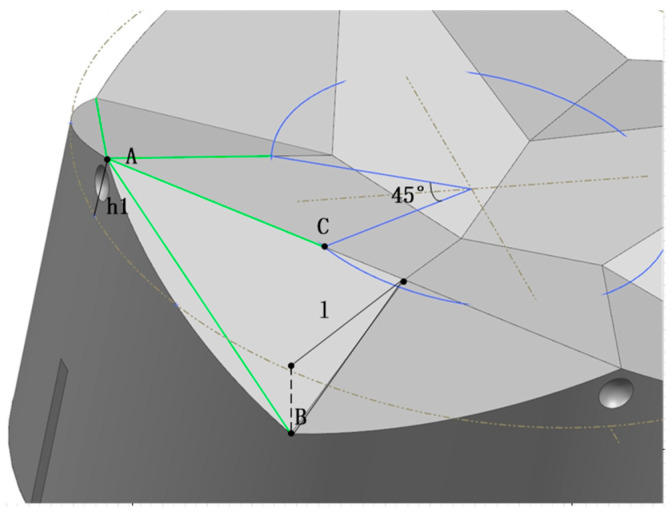
Inclination misalignment tolerance analysis model drawing.

The inclination misalignment MI between the two interfaces is shown in Figure 8d. For successful docking, the apex of the tapered top must be inserted into the corresponding hole of the opposite mechanism [17]. The minimum inclination MI for this inclination is shown in Figure 11:

MI = θ,D/2(1 − cosθ) + (h + h0)sinθ <= l(8)

## 4. Simulation Verification

Dynamics simulations of the docking interface were performed using Recur Dyn. Three simulation tests were carried out to verify the rotational, translational and inclination misalignment tolerances, respectively.

### 4.1. Simulation Parameter Settings

Based on the material and mounting space of the components, the simulation model parameters and contact constraints were initially determined and are shown in Table 1.

**Figure 12:**
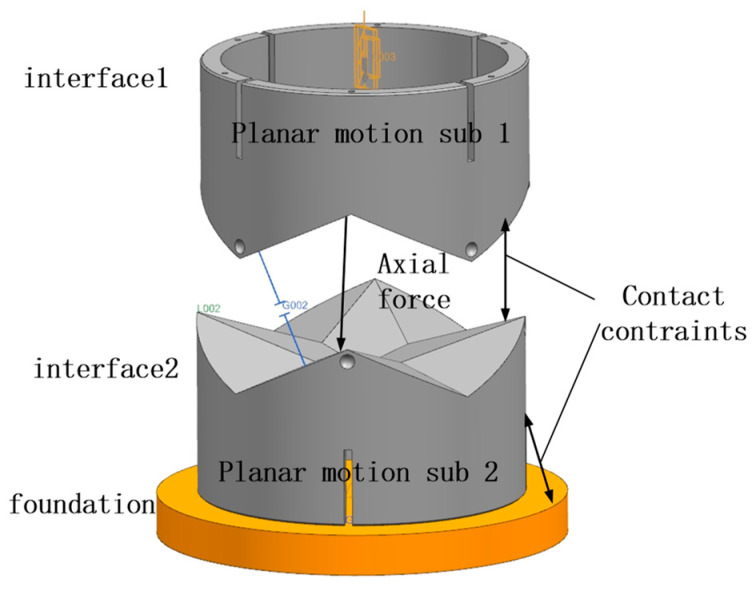
Docking simulation parameter models.

The locking motion is performed after alignment, so the effect of the mechanism on the misalignment tolerance could be ignored in the simulation. The docking simulation model is simplified, as shown in Figure 12, whereby interface 1 is actively docked and interface 2 adapts to interface 1 through motion to complete the docking. Interface 1 and interface 2 are each set up with a planar sub, and the two planes are orthogonal to accommodate the inclination misalignment tolerance. Interface 1 moves downward under a vector force, and contact constraints are set between interface 1 and interface 2. The specific parameters are shown in Table 1.

Simulations of rotational and translational misalignment were performed with the two interfaces parallel. Since the distribution of the four conical blocks is symmetric around the center, the maximum rotational misalignment tolerance was tested at 5° intervals over any 90° range when the axes of the two docking mechanisms coincide. Translational misalignment tolerances were simulated between the two axes in 1 mm translation increments [21]. The maximum camber misalignment tolerance was measured between the two interfaces in 1-degree increments. In the following, we analyze the simulation results of the measured maximum tolerance to determine whether the interface completed the docking by observing the coordinate displacement curves at the center of the mass position of the two interfaces.

### 4.2. Rotational Misalignment Tolerance Simulation

**Figure 13:**
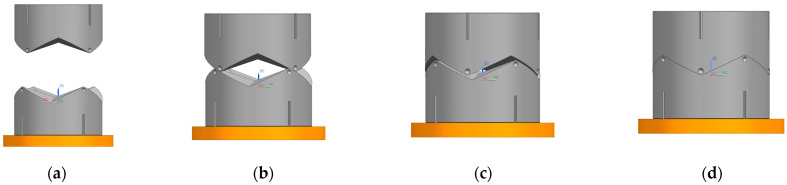
Rotational misalignment tolerance simulation process. (**a**) start; (**b**) touch; (**c**) amend; (**d**) success.

**Figure 14:**
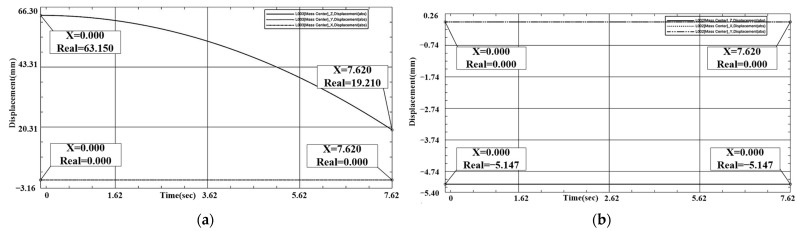
(**a**) Interface 1 rotational tolerance docking coordinate displacement plot. (**b**) Interface 2 rotational tolerance docking coordinate displacement plot.

In terms of parallel rotation misalignment tolerance, we know from previous analysis that a rotation in any angular range can lead to successful docking. The simulation charts the initial position to analyze and illustrate this. As shown in Figure 13 and Figure 14, X1 = Y1 = 0 mm, Z1 = 19.195 mm, X2 = Y2 = 0 mm, Z2 = −5.147 mm. During the docking process, interface 1’s downward movement comes into contact with interface 2. In the process, interface 2, only in-plane rotational movement, adapts to interface 1. Interface 1 of the X, Y coordinates does not change. The docking center of mass in the Z-direction distance is 24.342 mm, which meets the docking success criteria.

### 4.3. Simulation of Translational Misalignment Tolerance

**Figure 15:**
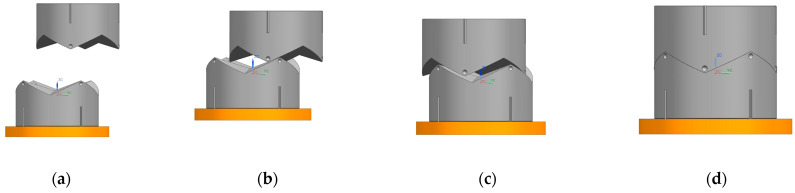
Translational misalignment tolerance simulation process. (**a**) start; (**b**) touch; (**c**) amend; (**d**) success.

**Figure 16:**
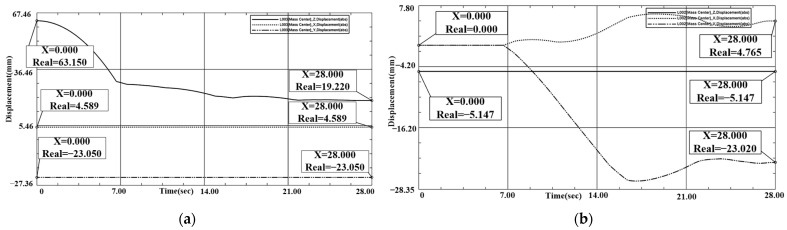
(**a**) Interface 1 translation tolerance docking coordinate displacement plot. (**b**) Interface 2 translation tolerance docking coordinate displacement plot.

In the simulation process of translational misalignment tolerance, the maximum misalignment tolerance simulation result graph was obtained, as shown in Figure 15 and Figure 16. The docking was judged to be successful or not by observing the center of mass coordinates of the interface movement displacement. When interface 1 contacts interface 2 during downward movement, interface 2 will move in the plane to adapt to interface 1, and finally the two become completely docked. The center of mass position coordinates of interface 1 were: X1 = 4.589 mm, Y1 = −23.05 mm, Z1 = 19.22 mm. X2 = 4.765 mm, Y2 = −23.02 mm, Z2 = −5.147 mm. X and Y coordinates coincided, and after docking the center of mass coordinates of interface 1 were aligned. The coordinates coincided, and the distance of the center of mass in the Z direction after docking was 24.367 mm, which meets the docking success criteria.

### 4.4. Camber Misalignment Tolerance Simulation

**Figure 17:**
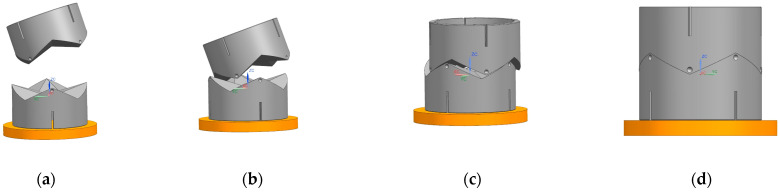
Inclination misalignment tolerance simulation process. (**a**) start; (**b**) touch; (**c**) amend; (**d**) success.

**Figure 18:**
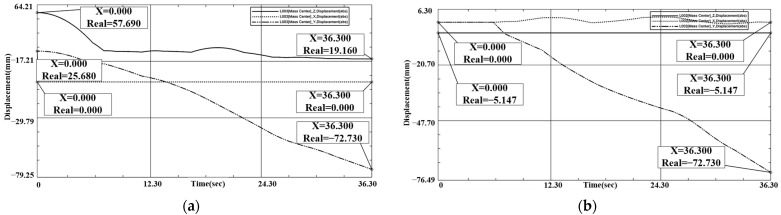
(**a**) Interface 1 inclination tolerance docking coordinate displacement plot. (**b**) Interface 2 inclination tolerance docking coordinate displacement plot.

In the simulation process of tilt misalignment tolerance, the simulation result diagram of tilt misalignment tolerance was obtained, as shown in Figure 17 and Figure 18. This determines whether the docking was successful or not by observing the displacement of the center of mass coordinate movements of the interface. When Interface 1 is in a downward tilt at a certain angle in the process of movement, and comes into contact with interface 2, interface 2 moves in the plane to adapt to interface 1 and finally the two become completely docked. Interface 1’s center of mass position coordinates were: X1 = 0 mm, Y1 = −72.73 mm, Z1 = 19.16 mm. X2 = 0 mm, Y2 = −72.73 mm, Z2 = −5.147 mm. After docking, the X and Y coordinates coincide and the distance of the center of mass in the Z direction after docking was 24.361 mm, which meets the docking success criteria.

### 4.5. Analysis of Simulation Results

The theoretical error tolerances can be calculated based on Equations (7) and (8). As shown in Table 2, the translational and angular misalignment tolerances obtained by calculation and simulation are approximate matches.

The analysis results show that when the axes of the two docking mechanisms coincide, successful docking can be established in any rotational misalignment. The translational misalignment tolerance can reach the minimum internal tangent circle radius of 23.5 mm, and the inclination misalignment tolerance can reach 24°.

## 5. Docking Experiment Validation

To further validate the simulation results, docking experiments were conducted for the three misalignment tolerances as well as the hexagonal lens splicing. During the experiment, the docking interface was fixed at the end of the robot arm, and a six-dimensional force/torque sensor was equipped between the end of the robot and the docking interface.

### 5.1. Misalignment Tolerance Experiment

**Figure 19:**
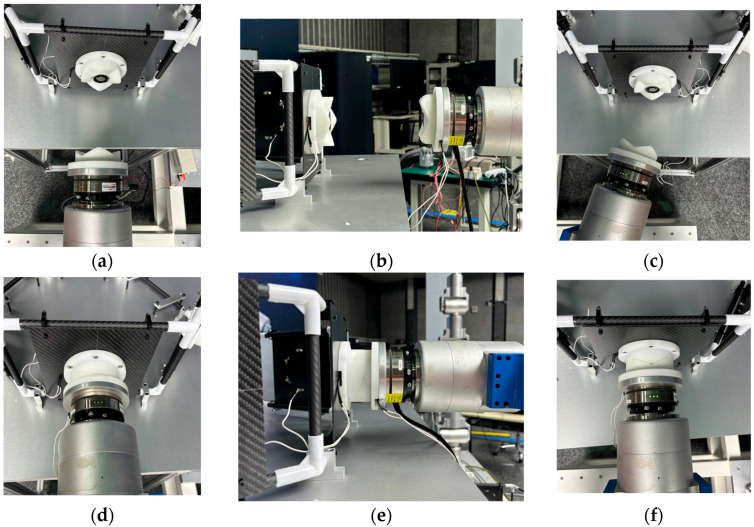
This is a figure. Schemes follow the same formatting. (**a**) Rotational tolerance; (**b**) Translational tolerance; (**c**) Inclination tolerance; (**d**) Rotational docking complete; (**e**) Panning docking complete; (**f**) Inclined docking complete.

Docking experiments were carried out with a translational misalignment tolerance of 23.5 mm, an inclination misalignment tolerance of 24° and a rotational misalignment tolerance of 90° due to the interface’s symmetrical structure, respectively. Docking was possible in all three cases, as shown in Figure 19.

The docking process determines the success of the docking by analyzing the value of the six-dimensional force/moment sensors to see if the docking was accurate or not. Ideally there is only docking force along the axial direction in the docking, the values of the six-dimensional force/moment sensors are shown in the figure.

**Figure 20:**
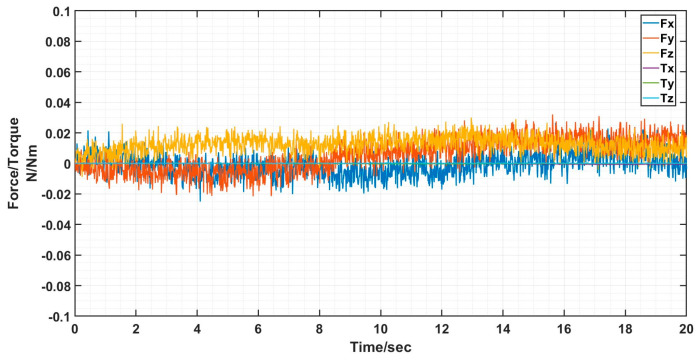
Schematic diagram of six-dimensional force/moment changes in rotational misalignment docking.

**Figure 21:**
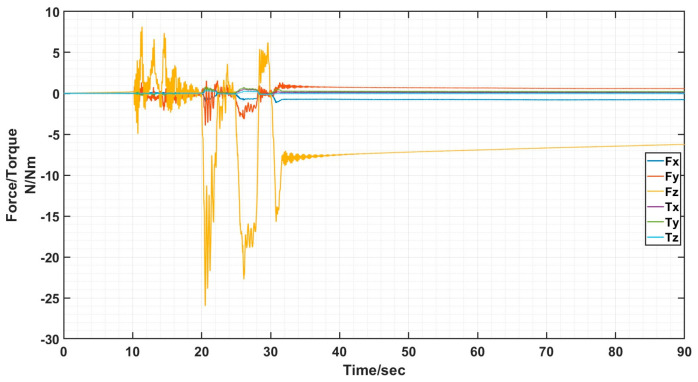
Schematic diagram of six-dimensional force/moment changes in translational misalignment docking.

**Figure 22:**
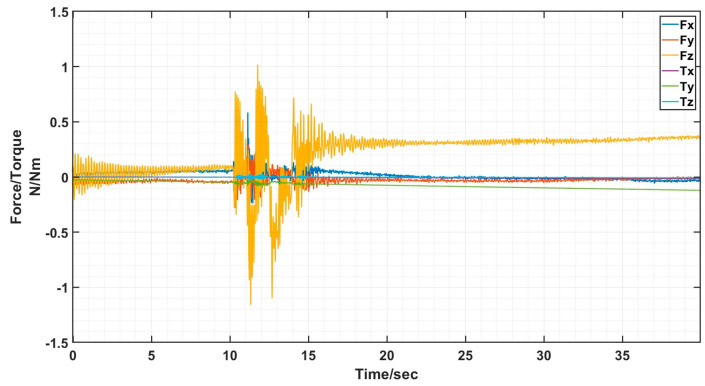
Schematic diagram of six-dimensional force/moment changes in inclination misalignment docking.

The interface docking was eventually only Fz axial force. The size of the axial force is related to the movement of the robot arm; the other direction of the force and moment is zero and can be ignored. The rotational misalignment tolerance experimental data showed that the value was stable near 0, with a maximum of 0.02 N and 0.02 Nm, which is negligible. The translational misalignment tolerance experimental data showed fluctuations in the process of movement. The Fz showed a maximum value of −25 N and eventually stabilized at −10 N or so; the other direction of force and moment was stable at near 0. The experimental data of inclination misalignment tolerance showed that there were fluctuations during the movement, with a maximum value for Fz reaching 1 N, which finally stabilized at about 0.5 N; the force and moment in other directions were stable at near 0. The results of the experimental data were all stable and convergent, indicating successful docking, as shown in Figure 20, Figure 21 and Figure 22.

### 5.2. Lens Splicing Experiment

**Figure 23:**
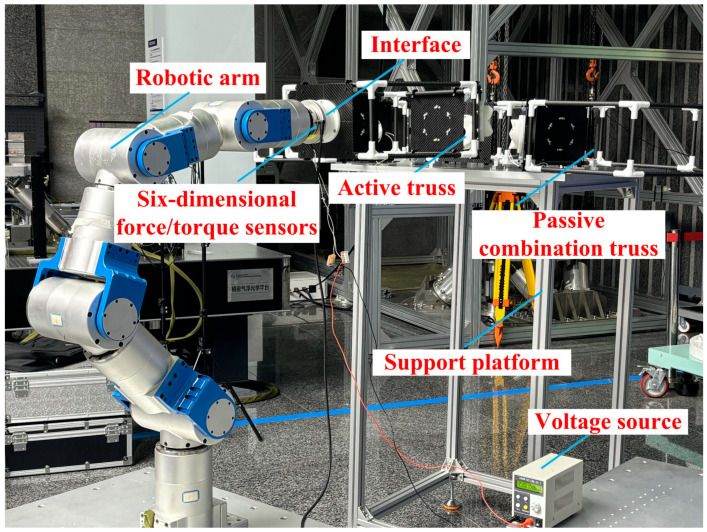
Illustration of truss splicing experiment with docking interface.

The misalignment tolerance capability of the docking interface was further verified through lens splicing experiments, where a robotic arm was used to clamp the hexagonal truss structure to simulate space telescope lens splicing, as shown in Figure 23.

The end of the robotic arm was equipped with docking interfaces, and the hexagonal truss was connected to the end of the robotic arm through two docking interfaces. The robotic arm clamped a hexagonal truss and combined it with three spliced trusses; the three interfaces were docked at the same time during the docking process. The docking process was evaluated by recording the changes in the six-dimensional force/torque sensors during the docking process.

**Figure 24:**
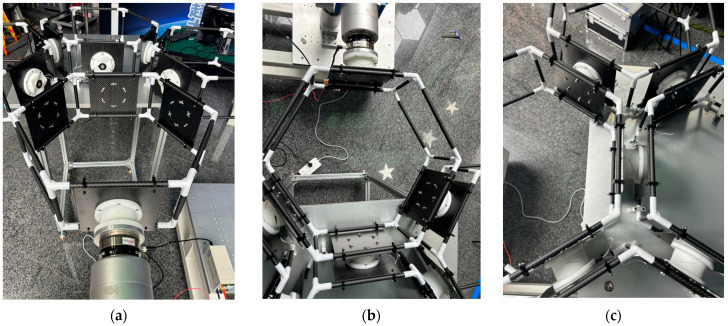
Schematic diagram of joist splicing process with docking interface (**a**) Start docking; (**b**) Amend procedure; (**c**) Successful docking.

**Figure 25:**
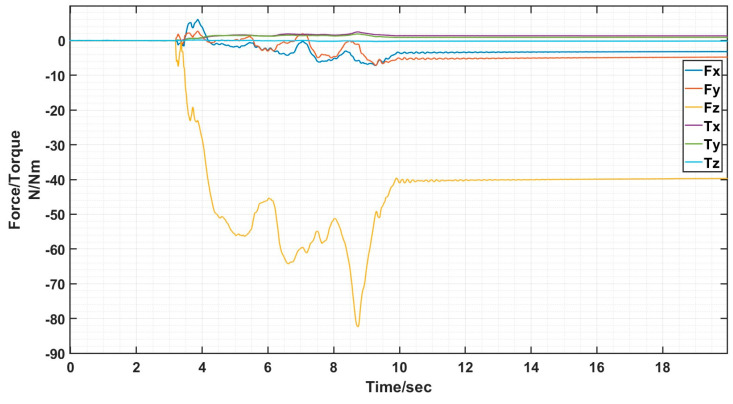
Schematic diagram of six-dimensional force/moment changes during truss splicing process.

When the truss was spliced, it would start to be spliced by gravity as well as vibration and collision during the stability of the movement process, and finally there was only Fz axial force, and the size of the axial force was related to the movement of the mechanical arm, and the force and moment in the other directions was zero, which can be neglected, as shown in Figure 24 and Figure 25.

## 6. Discussion

We derived the rotational misalignment tolerance as any angle, translational misalignment tolerance was 25 mm, and inclination misalignment tolerance was 28° through theoretical calculation.

In the docking simulation verification of misalignment tolerance, we analyzed the motion displacement graph of interface 1 and interface 2 to determine whether the docking was successful and whether the center distance between interface 1 and interface 2 is always at a constant value after docking has occurred. Through several simulations, the rotational misalignment tolerance was any angle, the translational misalignment tolerance was 23.5 mm, and the inclination misalignment tolerance was 24°. This was in line with our expectations.

In the interface misalignment tolerance docking experiment and six deformation truss splicing experiment, the values of six-dimensional force/torque sensors were used to analyze whether interface 1 and interface 2 docked successfully or not. Ideally, except for the axial force in the direction of the docking movement, the rest of the values should be as small as possible; ideally, they would be 0. Analyzing the resultant data, it was clear that the interface docked successfully.

The final verification showed that the tolerance of rotational misalignment was any angle, the tolerance of translational misalignment was 23.5 mm and the tolerance of inclination misalignment was 24°. That the interface could also be adapted to the task of hexagonal truss splicing was also verified.

## 7. Conclusions

This article proposed a new type of androgynous docking interface. The structure and working process of the locking mechanism of the docking interface was introduced to demonstrate its high efficiency and stable capability. The article also focused on analyzing the structural design of the docking surface of the interface, which can be docked with high efficiency and high tolerance by adopting an androgynous 90° symmetrical tapered block design.

Theoretical calculations and simulation results on misalignment tolerance showed that the docking mechanism had a self-alignment function, especially in terms of rotational error, that can accommodate the rotational misalignment tolerance at any angle. The interface also exhibited high translational and inclination misalignment tolerances. In this paper, the structural design and misalignment tolerance analyses of the docking interface were carried out, and the performance of the interface was verified through simulation and experiment to achieve the expected goal. The characteristics of this docking mechanism indicate its value for further research and its potential for application in modular space robotics.

In future work, the design of the locking mechanism will be further optimized to separate the motion during locking into axial movement and locking, which will result in a more compact structure and lower energy consumption. The space on-orbit service system will be built and system-level experiments will be conducted.

## Figures and Tables

**Table 1:** Simulation model parameters and contact constraints.

Parametric	Numeric	Unit
Mass	0.6607	kg
Stiffness factor	10,000	N/mm
damping factor	10	N-sec/mm
Friction factor	0.2	
Axial forces	1	N

**Table 2:** Calculated and simulated values of tolerance.

Misalignment	Rotational Tolerances (deg)	Translational Tolerance R_C_ (mm)	Inclination Tolerance (deg)
Theoretical value	Arbitrary angle	25	28
Actual value	Arbitrary angle	23.5	24

## Data Availability

The data presented in this study are available on request from the corresponding author.
